# Digital holographic microscopy for rapid bacteria segmentation and counting in microfluidic cartridges: basic considerations and limitations for diagnostic application

**DOI:** 10.1117/1.JBO.30.10.106501

**Published:** 2025-10-15

**Authors:** Hussein Kamel, Julian Schmid, Moaaz Rauf Nizami, Igor Alekseenko, Florian Hausladen, Daniel Claus, Rainer Wittig, Damien P. Kelly

**Affiliations:** Institut für Lasertechnologien in der Medizin und Messtechnik an der Universität Ulm (ILM), Ulm, Germany

**Keywords:** digital holographic microscopy, off-axis holography, bacteria, microfluidic, signal-to-noise ratio, numerical refocusing

## Abstract

**Significance:**

Digital holographic microscopy (DHM) has proven effective for particle segmentation within a given volume, making it well-suited for rapid monitoring of bacterial growth in microfluidic cartridges—such as in single-cell-based antimicrobial susceptibility testing assays. However, the development of optimal assays depends on a range of factors related to the instrument, consumables, and the sample itself. Despite this, comprehensive investigations into how these parameters influence the quality of the resulting phase images remain limited.

**Aim:**

To address this problem, we systematically explore the effect of these factors, including the microfluidic chamber height and its material properties, the density of the suspension, and other sample-inherent properties, on the signal-to-noise ratio (SNR) of the reconstructed phase image.

**Approach:**

We constructed an off-axis digital holographic microscope and defined a robust numerical processing pipeline allowing for the numerical reconstruction, refocusing and counting of suspended particles in a measurement volume spanning roughly $120\times 120\times 400\;\mu m^{3}$, at 50× magnification. We analyzed the performance of this system using various dilution steps of silica microspheres, Gram-positive spherical *Staphylococcus warneri* and Gram-negative rod-shaped *Escherichia coli* bacteria, filled in commercial microfluidic chips with different chamber heights.

**Results:**

Experimental results demonstrated the system’s capability in reflecting the dilution steps over 2 to 3 orders of magnitude. Our SNR analysis highlighted the microfluidic chamber height and the density of the suspension as key contributors to the background noise, whereas the particles themselves seemed to have a negligible effect. From this insight, we were able to derive an analytical function to predict the SNR of a given DHM system for various concentrations, chamber heights, and particle types.

**Conclusions:**

We successfully built a DHM system for counting suspended particles over a wide concentration range and for various microfluidic chamber heights. We also derived an initial framework for predicting and optimizing the performance of a given DHM system.

## 1 Introduction

Digital holographic microscopy (DHM) is a label-free phase imaging technique that can measure refractive index variations in a sample under inspection. DHM belongs to a group of analysis methods collectively referred to as quantitative phase imaging (QPI).^1^ An advantage of this approach is that a single hologram capture, followed by appropriate numerical processing, can be used to segment particles within a 3D volume.^2^^–^^5^ The single exposure-based approach theoretically qualifies DHM for rapid counting of bacteria in 3D volumes, thereby allowing for high-throughput cyclic monitoring of bacterial growth in microfluidic cartridges during, e.g., antimicrobial susceptibility testing (AST). However, the successful realization of a DHM-based AST assay requires the optimization of several instrument-, consumable-, and sample-related parameters that affect the signal-to-noise-ratio (SNR). These include (1) the density of the cell suspension and accumulation of cells in the axial direction, (2) microfluidic chip properties such as material homogeneity and surface roughness, and (3) maximum axial extension of fluidic chambers that may be dictated by mass production requirements. There are a few reports that investigate the feasibility of using QPI for the analysis of particle and cell suspensions, as well as immobilized single bacteria and bacterial colonies.^6^^–^^8^ However, systematic investigations concerning the impact of the parameters described above on the SNR are lacking. In this study, we used DHM to characterize differentially concentrated suspensions of microspheres as well as model bacteria (rod-shaped Gram-negative *Escherichia coli* and spherical Gram-positive *Staphylococcus warneri*) using a self-constructed DHM device and a collection of published reconstruction/analysis algorithms to address the following questions:

1. How do the channel height and material properties of the microfluidic device, the density of the suspension, and other sample properties, such as size and shape, affect the counting results?
2. Is it possible to analytically relate the parameters mentioned in 1 to quantitatively predict a concentration range that can be measured with a given DHM system?

Our results will provide support for scientists who intend to develop DHM-based assays for the quantitation of particle or bacteria suspensions, e.g., for antibiotic susceptibility testing in microfluidic cartridges. A major contribution of this paper is to present a robust numerical processing pipeline to resolve and count small particles suspended in microfluidic chambers. In this numerical processing pipeline, we found several steps to be of significant importance:

1. Filtering with a soft-edged aperture—in our case, using a super-Gaussian.^9^
2. Zernike-based aberration correction.^10^^,^^11^
3. We also use a technique proposed by Nagahama^12^—entitled the “ringing artifact extraction method”—where we attempt to compensate for ringing artifacts that arise from diffraction when we refocus our wavefield through the microfluidic volume.

Using these three processing steps, we have found that we can significantly improve the SNR in our single-shot holographic capture.

## 2 Methods

### 2.1 Off-axis Digital Holographic Microscopy

**Fig. 1:**
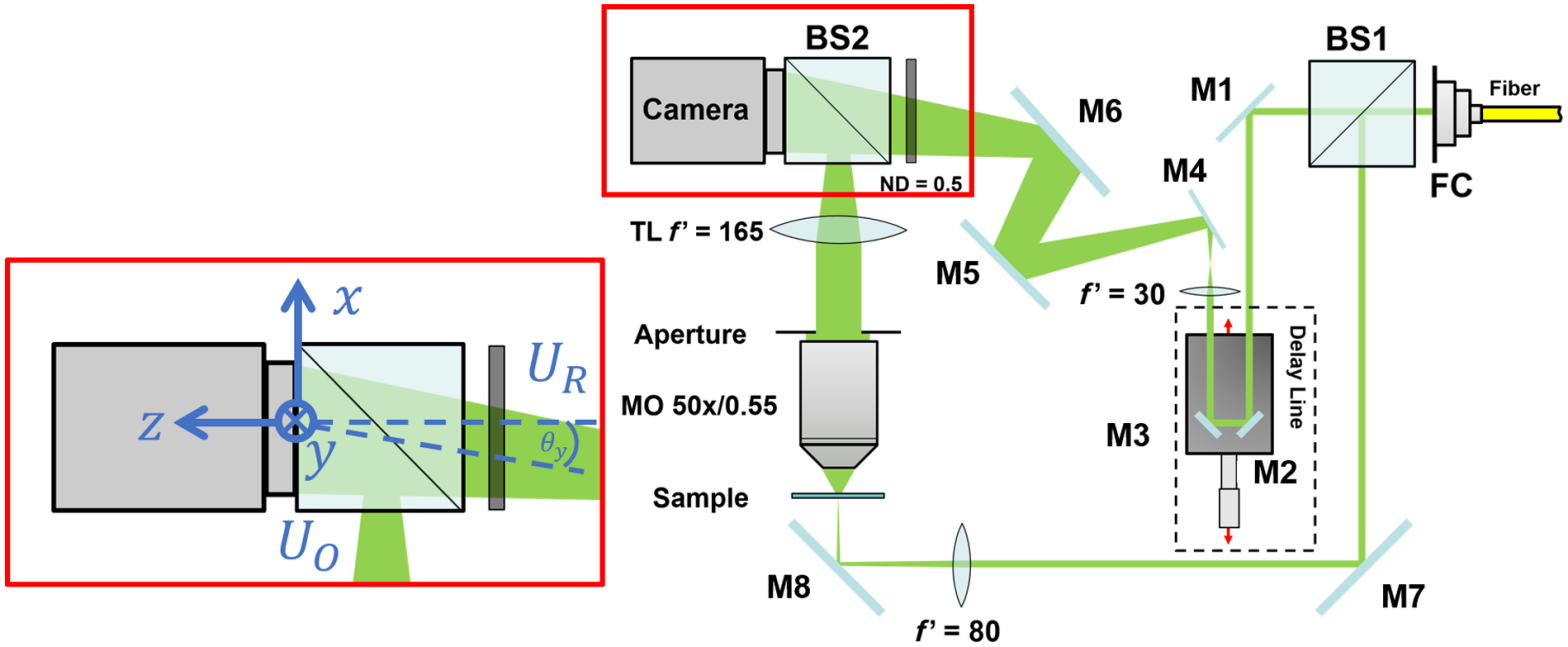
Schematic view of the proposed off-axis DHM with an enlarged view showing the coordinate system. The focal lengths of the lenses are specified in millimeters. M, mirror, BS, beam splitter; MO, microscope objective; TL, tube lens; FC, fiber coupler; ND, neutral density filter; $U_{O}$, object wavefield; $U_{R}$, reference wavefield; and $\theta_{y}$, tilt angle of the reference wavefield $U_{R}$.

In this work, we employ an off-axis DHM, as shown in Fig. 1, for the quantitative phase imaging of the sample. To achieve twin-image-free imaging and separate the real image from the virtual image, we make use of an off-axis reference wave, as proposed by Leith and Upatnieks.^13^ The hologram of the interference between the object wavefield $U_{O}$ and the off-axis reference wavefield $U_{R}$ is captured and numerically processed, as described by Cuche et al.,^9^ to filter out the real image term. In this case, we used a higher order elliptical Gaussian function^14^—also known as a super-Gaussian function—as a filter to avoid high-frequency fluctuations in the reconstructed image.^9^ This filtering results in a twin-image-free object wavefront $U_{O}$, which can be further processed to reconstruct its intensity and phase ϕ. The reconstructed phase will not have an ideal flat phase distribution and will suffer from (1) residual linear phase component due to the off-axis arrangement; (2) residual curvature or higher order aberrations, caused by the optical components in the system and how they are laid out. This very point is discussed at length by Sánchez-Ortiga et al.^15^ It was shown that certain DHM imaging setups leave a residual curvature of the object wavefield. In our setup, we attempt to correct for this by allowing the reference wave to diverge slightly, as discussed in detail in previous publications.^16^^–^^18^ From experiments, we have adjusted the lens in the reference arm in its focal length and position to achieve a relatively flat phase. Any residual distortions in the phase can often be compensated for using a calibration procedure.^19^ However, in this work, we will make use of a numerical compensation algorithm to mitigate the contribution of any aberration originating from the microfluidic chamber and its positioning. For this, the reconstructed object wavefront is first multiplied by a wavefront of opposite tilt to the reference wave. This compensates for any residual tilt arising from the off-axis configuration and sample positioning. Then, the phase is unwrapped^20^ and decomposed onto a Zernike basis^21^ with a sufficient number of Zernike terms to construct an aberrations mask, which is subsequently subtracted from the unwrapped phase.^10^^,^^11^ The final result is an aberration-corrected wavefield. To examine the distribution of bacteria or particles within a 3D volume, we then propagate this corrected wavefield to a set of planes within the microfluidic chamber using the angular spectrum method.^22^^–^^24^ However, when we do this, we have observed ringing artifacts in the phase distribution that arise due to the diffraction process. These ringing artifacts can lead to problems with our segmentation and counting algorithm. To minimize these effects, we consider the “ringing artifact extraction method” algorithm proposed by Nagahama.^12^ Nagahama considers propagating a constant phase distribution to each plane of interest in the microfluidic chamber. The ringing artifacts that arise from diffraction are also present in each of these distributions. Following the description in Ref. 12, we subtract the phase of each calculated idealized distribution from the reconstructed phase distribution of our samples. The algorithm runs as follows: For a given reconstruction plane, we propagate our corrected wavefield by the appropriate value for z; we also calculate the idealized phase distribution for the same value of z (assuming a constant phase input) and subtract the phase signals—thereby reducing diffraction artifacts.

### 2.2 Counting Algorithm

After propagating the wavefront to the different layers and calculating the phase, the phase volume can now be segmented for bacteria, which is done using a basic thresholding algorithm and an empirically determined thresholding value. We work under the assumption that when a bacterium is in focus, we will observe the most significant deviation in the phase profile as is shown in Ref. 18 and later in the experimental results presented in Sec. 4. Before performing a connected component analysis on the binary volume, the volume is morphologically opened using a volumetric structuring element of similar shape and size to the structures to be counted (cube-shaped for rod-shaped bacteria and spherical in the case of spherical bacteria).^25^ Finally, the volume of each segmented structure is determined and used to statistically filter out segmented regions, which do not fit in an expected distribution centered around the average volume measured among the segmented binary structures. To account for clustered cells, the count is calculated by dividing the total volume of each segmented structure by the average volume of a single cell.

## 3 Experimental Setup

### 3.1 Optical Setup

The optical setup shown in Fig. 1 uses a fiber-coupled laser (Cobolt 06-MLD 520 nm) from Hübner Photonics with a bandwidth of ca. 1 nm and a coherence length of ca. 200  μm. The beam is collimated and split into two parts: an object beam and a reference beam. The object beam is focused down onto the bacteria sample and imaged using a microscope objective (LD EC Epiplan-Neofluar 50×/0.55) from Zeiss. The magnified image after the objective is then relayed onto the camera sensor (Manta G-235B) from Allied Vision using a tube lens (TTL 165-A) from Thorlabs, Newton, New Jersey, United States. The reference beam is tilted using multiple mirrors to achieve the required off-axis separation in the Fourier domain while preventing the beam from clipping. Before tilting the reference beam, the beam is passed through a delay line, allowing for accurate adjustment of the optical path length, and diverged slightly using a bi-convex lens. Focusing the object beam down onto the sample, together with the use of a neutral density filter in the reference arm, allows us to balance the power of the two beams, thereby optimizing the interference contrast on the CMOS sensor. Due to low coherence of the laser source and the off-axis angle of the reference beam, the interference contrast on the CMOS sensor diminishes with increasing distance from the central region, limiting the usable measurement area to about $120\times 120\;\mu m^{2}$ on the microfluidic chip. The captured holograms were numerically processed and counted for bacteria, as described in Sec. 2, using MATLAB.

### 3.2 Sample Preparation

Bacterial suspensions [rod-shaped Gram-negative *Escherichia coli* (*E. coli*) strain K12 DH5-alpha (Invitrogen, Karlsruhe, Germany), spherical Gram-positive *Staphylococcus warneri* (*S. warnri*) strain DSM20316 (DSMZ, Braunschweig, Germany)] were grown overnight during shaking at 37°C in LB-medium (*E. coli*) or TSB-medium (*S. warneri*), respectively. The suspensions were adjusted to optical density ${\mathrm{OD}}_{600}=1$, and dilutions thereof were prepared in respective growth media. In the following text and figures, we refer to the dilutions by writing 1:x, where x indicates the respective dilution factor of the ${\mathrm{OD}}_{600}=1$ adjusted suspension. Correlating the actual bacterial count with the optical density depends on a multitude of physiological factors such as the growth phase of an overnight culture, rate of dead cells, and debris. Therefore, approximations were used. For *S. warneri*, an ${\mathrm{OD}}_{600}$ of one was estimated to correspond to a concentration of $∼7\times 10^{8}\;\text{cells}/\mathrm{mL}$, which was based on own colony forming unit (CFU) measurements (not shown) and published data concerning the related strain *Staphylococcus aureus*.^26^ For *E. coli*, we calculated with $5\times 10^{8}\;\text{cells}/\mathrm{mL}$ at ${\mathrm{OD}}_{600}=1$, basing on own CFU measurements and published data.^27^ DHM measurements were performed in microfluidic channels [channel height 200  μm: smart slide (#80816), channel height 400  μm: μ-slide VI flat (#80626), ibidi, Munich, Germany]. Subsequent to bacteria filling, channels were sealed and immediately imaged to prevent sedimentation.

In addition to measuring the concentrations of bacterial suspensions, this work aims to evaluate the scattering behavior of the analyzed samples. This is achieved using a standardized nonbiological sample resembling the bacteria as a base model, such as microspheres, which furthermore allow for the accurate calibration of the system behavior and isolation of certain dynamics. Monodisperse silica microspheres ${\mathrm{SiO}}_{2}$-F-SC117-2 from microParticles GmbH (Berlin, Germany) were analyzed. Given their average diameter of 0.985  μm, density of $1.85\;g/{\mathrm{cm}}^{3}$ and concentration of 5% w/v, the undiluted suspension originally contained around $5.405\times 10^{10}\;\text{particles}/\mathrm{mL}$, assuming an ideal spherical shape for each particle. Suspensions containing these microspheres were imaged using the same microfluidic chambers mentioned earlier and diluted down further using distilled water.

### 3.3 Measurement Procedure

Using the optical setup described in Sec. 3.1 and the processing pipeline detailed in Sec. 2, initial experiments using microspheres and bacteria (*E. coli* and *S. warneri*) were carried out. The measurements were performed using two different chamber heights by capturing an off-axis hologram at around the midpoint of the microfluidic chamber height and subsequently refocusing over a distance of ±125  μm (200  μm chamber height) and ±225  μm (400  μm chamber height), with a step size of 0.5  μm. The measured concentration is defined as the number of counted microspheres/bacteria divided by the measurement volume, which was ∼2.88  nL and 5.76 nL for the 200 and 400  μm variant, respectively. In the case of the microspheres, the analysis included three independent dilution series down to a dilution of 1:8000. For the bacteria, at least three biological replicates were considered down to a dilution of 1:100 of the ${\mathrm{OD}}_{600}=1$ adjusted suspension. The statistical results also included multiple holograms taken at different positions in the same microfluidic chamber. Please note that phase reconstructions of high-density suspensions exhibiting phase unwrapping issues (finite phase jumps) have been discarded from the statistics. This sometimes occurred at a dilution of 1:20 for the microspheres and a concentration of ${\mathrm{OD}}_{600}=1$ for the bacteria.

## 4 Results

### 4.1 Evaluation of Optical Setup with USAF Resolution Chart

**Fig. 2:**
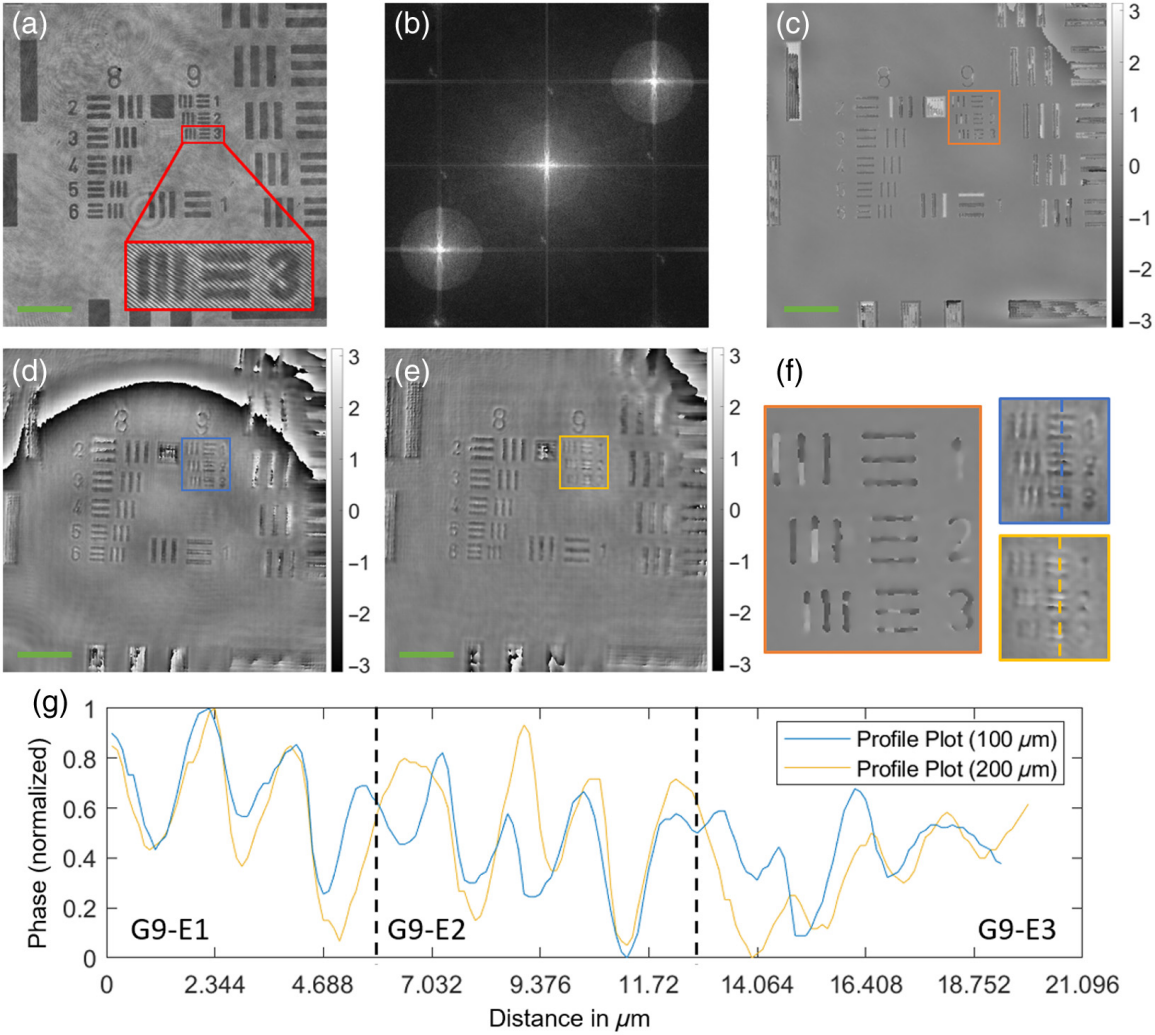
Evaluation of DHM system using a USAF resolution chart: (a) hologram of in-focus USAF resolution chart; (b) Fourier transform of the hologram in panel (a); (c) reconstructed phase image of the hologram in panel (a); (d) numerically refocused phase image of hologram taken 100  μm out-of-focus; (e) numerically refocused phase image of hologram taken 200  μm out-of-focus; (f) color-coded enlarged views of the marked regions in panels (c), (d), and (e); (g) color-coded profile plots along the dashed lines in panel (f). Green colored scale bars in panels (a), (c), (d), and (e) correspond to 20  μm. Please note that the spherical ringing artifacts seen in panels (d) and (e) originate from the used phase unwrapping algorithm.

The performance of the proposed DHM system has been evaluated using a USAF resolution target, as is seen in Fig. 2 and discussed in Ref. 28. In the case of an in-focus USAF chart, the system was capable of resolving structures down to group 9, element 3 (G9-E3) corresponding to a line width of 0.78  μm [Fig. 2(f)]. We then deliberately placed the USAF chart a distance of 200  μm from the in-focus plane and numerically refocused the reconstructed distribution. In this case, we could only resolve G9-E2 with a line width of 0.87  μm [Fig. 2(g)]. Thus, we can confirm a resolution of at least 1  μm over an entire numerical refocusing distance of 200  μm.

### 4.2 Counting Results with Microspheres

**Fig. 3:**
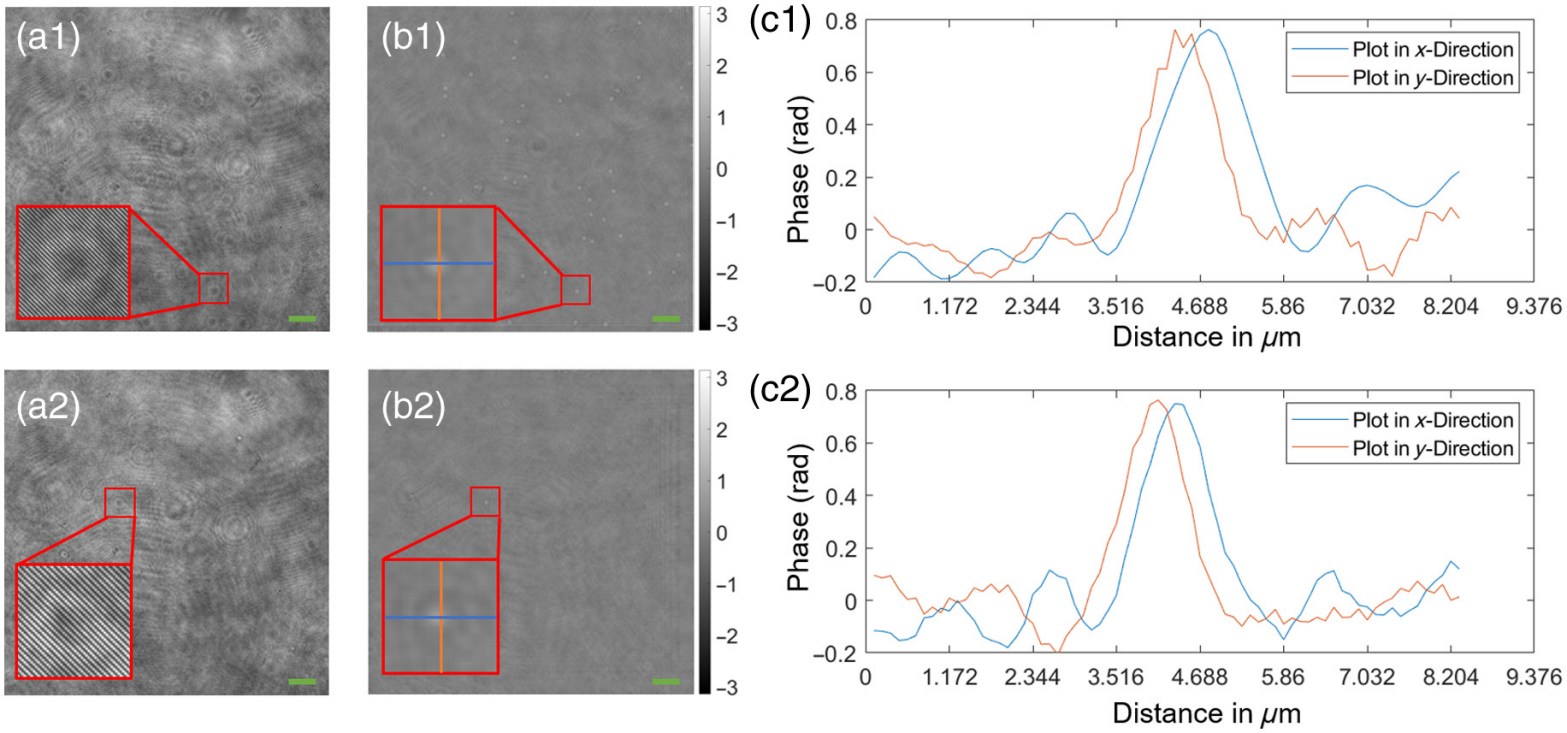
Evaluation of the DHM system using suspended microspheres, green colored scale bars correspond to 10  μm. Rows (1) to (2) microspheres at 200 and 400  μm chamber height and a dilution of 1:2400 and 1:8000, respectively. First column (a1–a2): recorded holograms with a magnified view of microspheres 10 and 30  μm out-of-focus, respectively; second column (b1–b2): numerically refocused phase images of holograms in column (a1–a2) with a magnified view at the same position as in (a1–a2); and third column (c1–c2): color-coded profile plots along the lines in column (b1–b2).

**Fig. 4:**
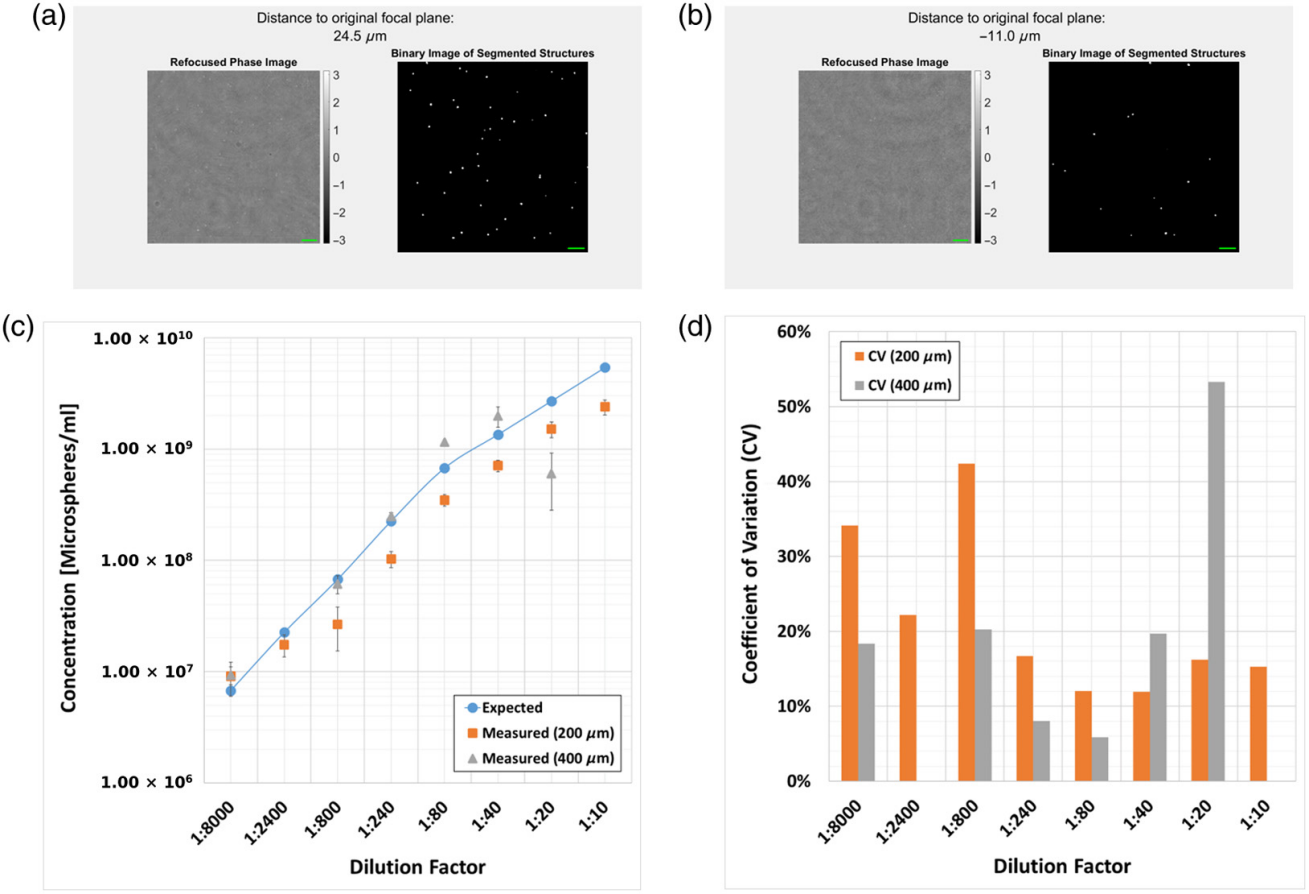
Numerical refocusing and counting results using microspheres. Representative images of videos showing the numerically refocused phase image (left view) and segmented structures (right view) for a 200  μm (a, Video 1) and 400  μm (b, Video 2) chamber height and a concentration of $6.76\times 10^{7}\;\text{microspheres}/\mathrm{mL}$. Refocused phase images and binary images of segmented structures represent identical fields of views for direct comparison, respectively. Green colored scale bars correspond to 10  μm. (c) Counting results for a 200 and 400  μm chamber height and a concentration range between $6.76\times 10^{6}$ and $5.41\times 10^{9}\;\text{microspheres}/\text{mL}$. Dilution factors on the x-axis refer to the original microsphere suspension containing $5.405\times 10^{10}$. Counting results include three independent dilution series, each taken at multiple positions within the same microfluidic chamber. The error bars represent one standard deviation of uncertainty. The dilutions 1:2400 and 1:10 for the 400  μm chamber height were omitted due to technical reasons; (d) coefficient of variation (CV) in percent, calculated as the ratio of the standard deviation to the mean particle count from the results shown in panel (c) (Video 1, MP4, 6.7 MB [URL: https://doi.org/10.1117/1.JBO.30.10.106501.s1]; Video 2, MP4, 13.3 MB [URL: https://doi.org/10.1117/1.JBO.30.10.106501.s2]).

Representative holograms of out-of-focus microspheres can be seen in Fig. 3, which, after numerical refocusing, were found to exhibit an average phase value of around 0.75 rad. Videos demonstrating the numerical refocusing and segmentation algorithms together with the counting results for various concentrations are presented in Fig. 4. It was found that the counting algorithm faithfully reflected the trend in the concentrations levels for both 200 and 400  μm chamber heights, resulting in inter-experimental coefficient of variation (CV) of up to 20% CV for most of the dilution factors—see Fig. 4(d). The deviations for both bacterial species under investigation were higher [see Secs. 4.3, 4.4 and Figs. 6(g)–6(h)], which may be related to sample-specific parameters such as size, shape, and refractive index, as well as inconsistencies/inaccuracies in the ${\mathrm{OD}}_{600}$ measurements.

### 4.3 Counting Results with Bacteria (*E. coli* and *S. warneri*)

**Fig. 5:**
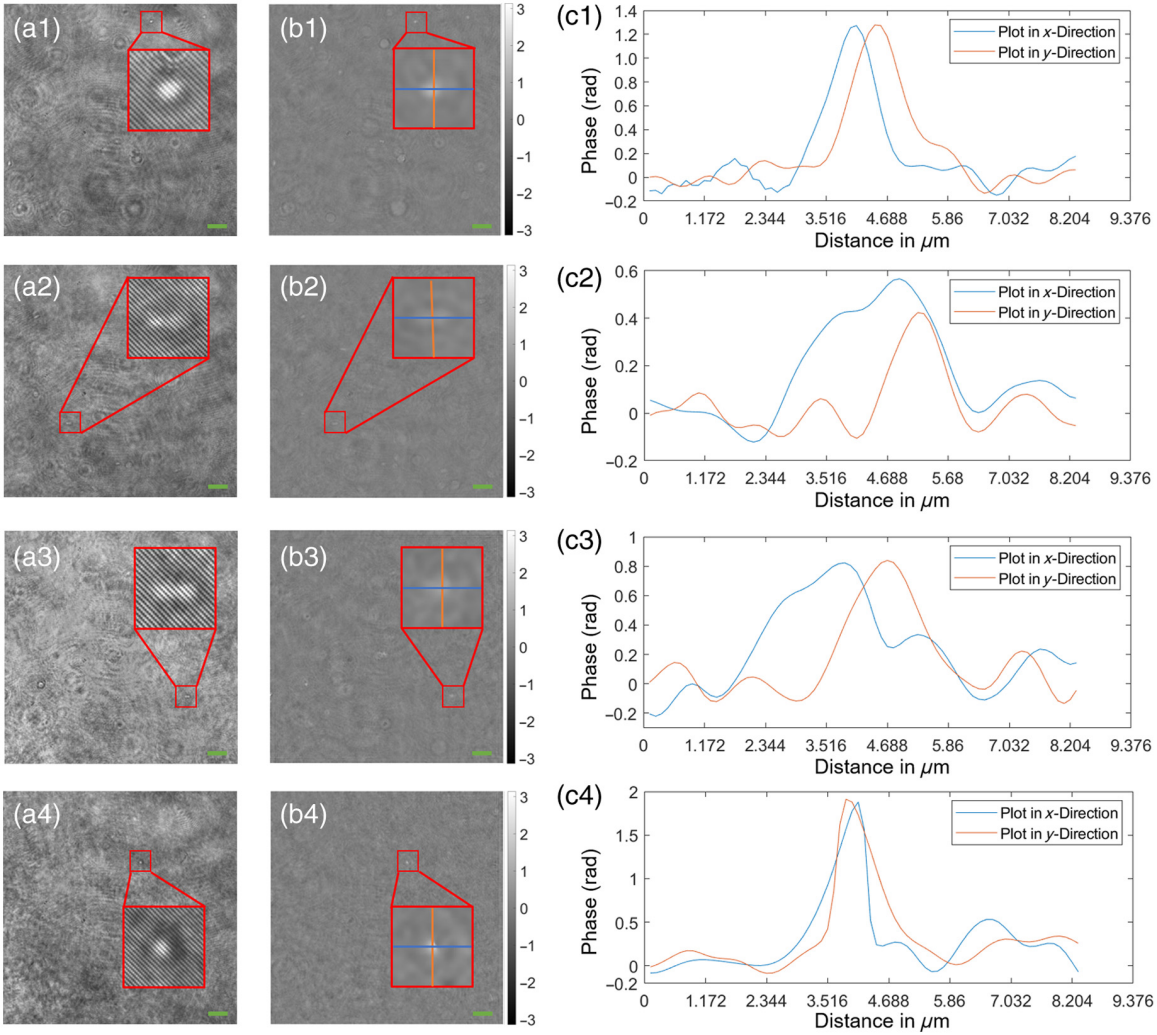
Evaluation of the DHM system using bacterial suspensions (1:10 dilution of ${\mathrm{OD}}_{600}=1$), green colored scale bars correspond to 10  μm. Rows (1) to (2) *S. warneri* and *E. coli*, respectively, at 200  μm chamber height and rows (3) to (4) *S. warneri* and *E. coli*, respectively, at 400  μm chamber height. First column (a1–a4): recorded holograms with a magnified view of bacteria 4 to 10  μm out-of-focus. Please note that in the case of *S. warneri*, two bacteria are observed to be aligned axially (a1) and transversely (a3); second column (b1–b4): numerically refocused phase images of holograms in column (a1–a4), respectively, with a magnified view at the same position as in (a1–a4); third column (c1–c4): color-coded profile plots along the lines in column (b1–b4).

**Fig. 6:**
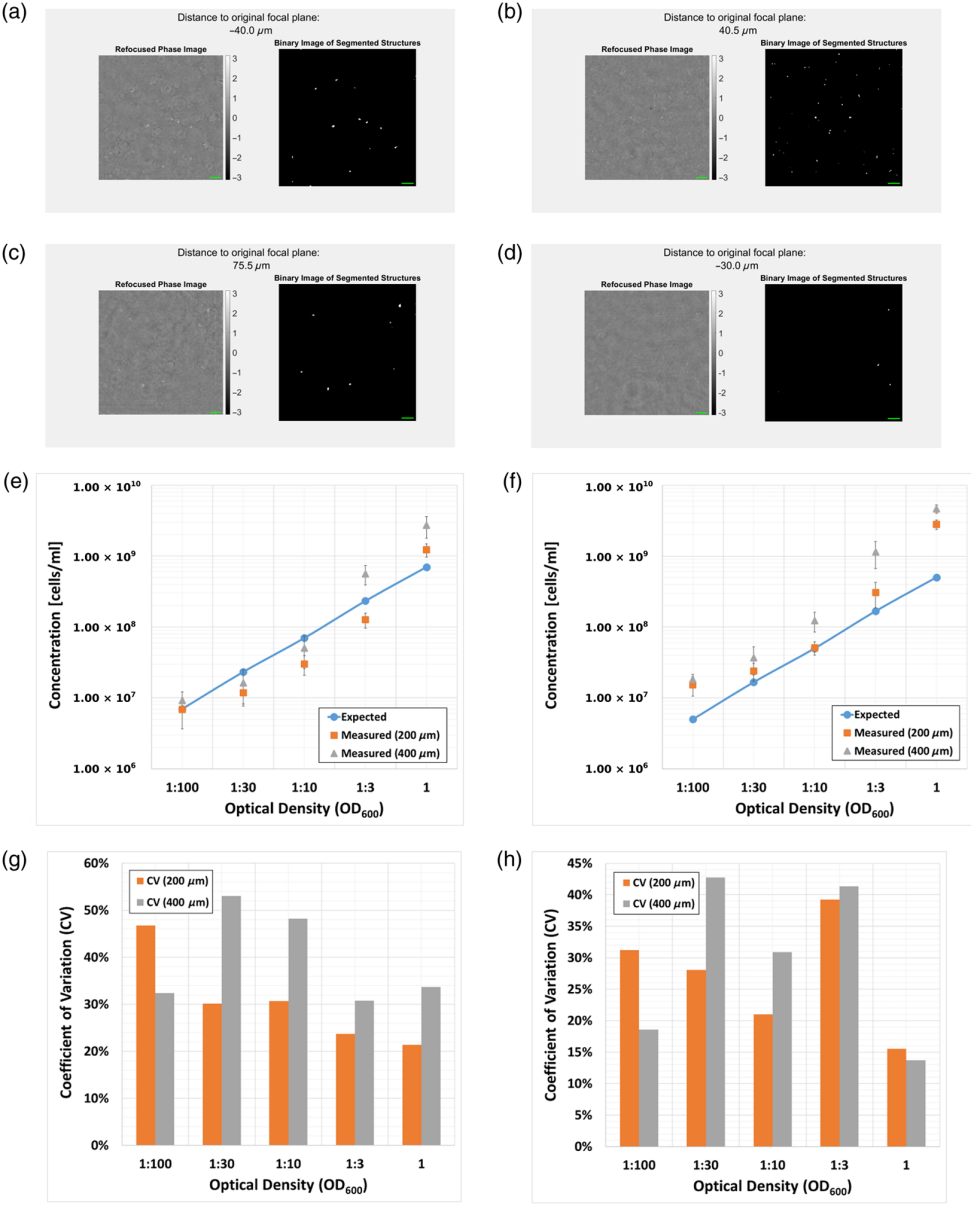
Numerical refocusing and counting results using bacteria. Representative images of videos showing the numerically refocused phase image (a) and segmented structures (b). (a) *S. warneri* at 200  μm and 1:3 dilution of an ${\mathrm{OD}}_{600}=1$ suspension (Video 3); (b) *E. coli* at 200  μm and 1:3 dilution of an ${\mathrm{OD}}_{600}=1$ suspension (Video 4); (c) *S. warneri* at 400  μm and 1:10 dilution of an ${\mathrm{OD}}_{600}=1$ suspension (Video 5); (d) *E. coli* at 400  μm and 1:30 dilution of an ${\mathrm{OD}}_{600}=1$ suspension (Video 6). Refocused phase images and binary images of segmented structures represent identical fields of views for direct comparison, respectively. Green colored scale bars correspond to 10  μm; (e) counting results for *S. warneri* at 200 and 400  μm chamber height; (f) counting results for *E. coli* at 200 and 400  μm chamber height. Dilution factors on the x-axis refer to a bacterial suspension adjusted to ${\mathrm{OD}}_{600}=1$. Counting results include at least three biological replicates, each taken at multiple positions within the same microfluidic chamber. The error bars represent one standard deviation of uncertainty; (g) and (h) coefficient of variation (CV) in percent, calculated as the ratio of the standard deviation to the mean particle count from the results shown in panels (e) and (f), respectively (Video 3, MP4 [URL: https://doi.org/10.1117/1.JBO.30.10.106501.s3], 7.83 MB; Video 4, MP4, 7.37 MB [URL: https://doi.org/10.1117/1.JBO.30.10.106501.s4]; Video 5, MP4, 12.8 MB [URL: https://doi.org/10.1117/1.JBO.30.10.106501.s5]; Video 6, MP4, 10.8 MB [URL: https://doi.org/10.1117/1.JBO.30.10.106501.s6]).

Representative phase images of numerically refocused bacteria are seen in Fig. 5. The cross-sectional plots along the color-coded lines show different phase values ranging between 0.6 and 2 rad, depending on the type and orientation of the bacteria to the optical axis. In rows 1 and 4 of Fig. 5, the bacteria are oriented parallel to the optical axis and exhibit higher phase values than their counterparts in rows 2 and 3, which are oriented perpendicular to the optical axis. Note that in Fig. 5(a1) and Fig. 5(a3), two *S. warneri* bacteria are observed to be aligned in axial and transversal direction, respectively. In Fig. 6, representative videos of the refocusing and segmentation algorithm can be seen together with the measured concentrations plotted, as cells/mL against the dilution factor of the original ${\mathrm{OD}}_{600}=1$ bacterial suspension. The concentrations measured showed a clear increase with denser media for both 200 and 400  μm chamber heights, reflecting the hypothetical cell counts of an ideal measurement quite well and differing only at the extremes of the optical density range. However, the bacterial concentrations measured for a 400  μm chamber height resulted in a higher detected cell count compared with the 200  μm variant, which was observable in both *E. coli* and *S. warneri*, with *E. coli* showing slightly stronger deviations from the expected measurement curve, especially at very low concentrations.

### 4.4 Analysis of Signal-to-Noise Ratio

As described in Sec. 1, we aim to develop a quantitative model for predicting the SNR of a given DHM system when measuring suspended microspheres/bacteria. Having prior knowledge of the SNR provides a reliable estimate of the system’s limitations and enables optimal dimensioning of key parameters—such as maximum concentration, chamber height, and wavelength—to better address the specific requirements of a given problem. In this section, we turn our attention to deriving this SNR model. Looking back at Figs. 4 and 6, the counting accuracy and phase image quality seem to diminish with increasing concentration and chamber height. Moreover, these effects seem to vary between sample types, indicating an underlying influencing factor. To better understand these effects, we will analyze how the background noise level varies depending on the different concentrations, chamber heights, and sample types. After numerical correction, the refocused phase ϕ can be expressed as the sum of two components: (1) the sample phase $\phi_{S}$ and (2) and a noise term $\phi_{N}$. In the previous analysis, we intentionally ignored the contribution of $\phi_{N}$; now, however, we investigate this term further, and in particular how it can affect the counting accuracy of a DHM system. First, we note that there are two types of noise sources making up $\phi_{N}$:

1. Base-level phase noise $\phi_{N0}$, which is always present in the optical system and is caused by certain sources such as the surface roughness and material inhomogeneity of the microfluidic chamber, dust particles on the optics, and back reflections. In other words, this type of noise is independent of the imaged particles (microspheres or bacteria).
2. Additional phase noise $\phi_{NS}$ caused by the scattering of the particles in the measurement volume. This type of noise is dependent on the type of imaged scatterers (microspheres or bacteria), their concentration, and the height of the chamber they occupy.

To quantify the background phase noise intensity $\phi_{N}$ of the entire measurement volume for a given sample type, chamber height, and concentration, we introduce the mean phase noise intensity $\left\langle \phi_{N}\right\rangle$ given by

$$\langle \phi_{N}\rangle =\frac{1}{N_{z}}\overset{z_{\text{max}}}{\underset{z}{\sum }}\{\sqrt{\frac{\overset{N_{xy}}{\underset{x,y}{\sum }}[\phi_{z}(x,y)-\langle \phi_{z}\rangle {]}^{2}}{N_{xy}}}\}.$$ (1)

**Fig. 7:**
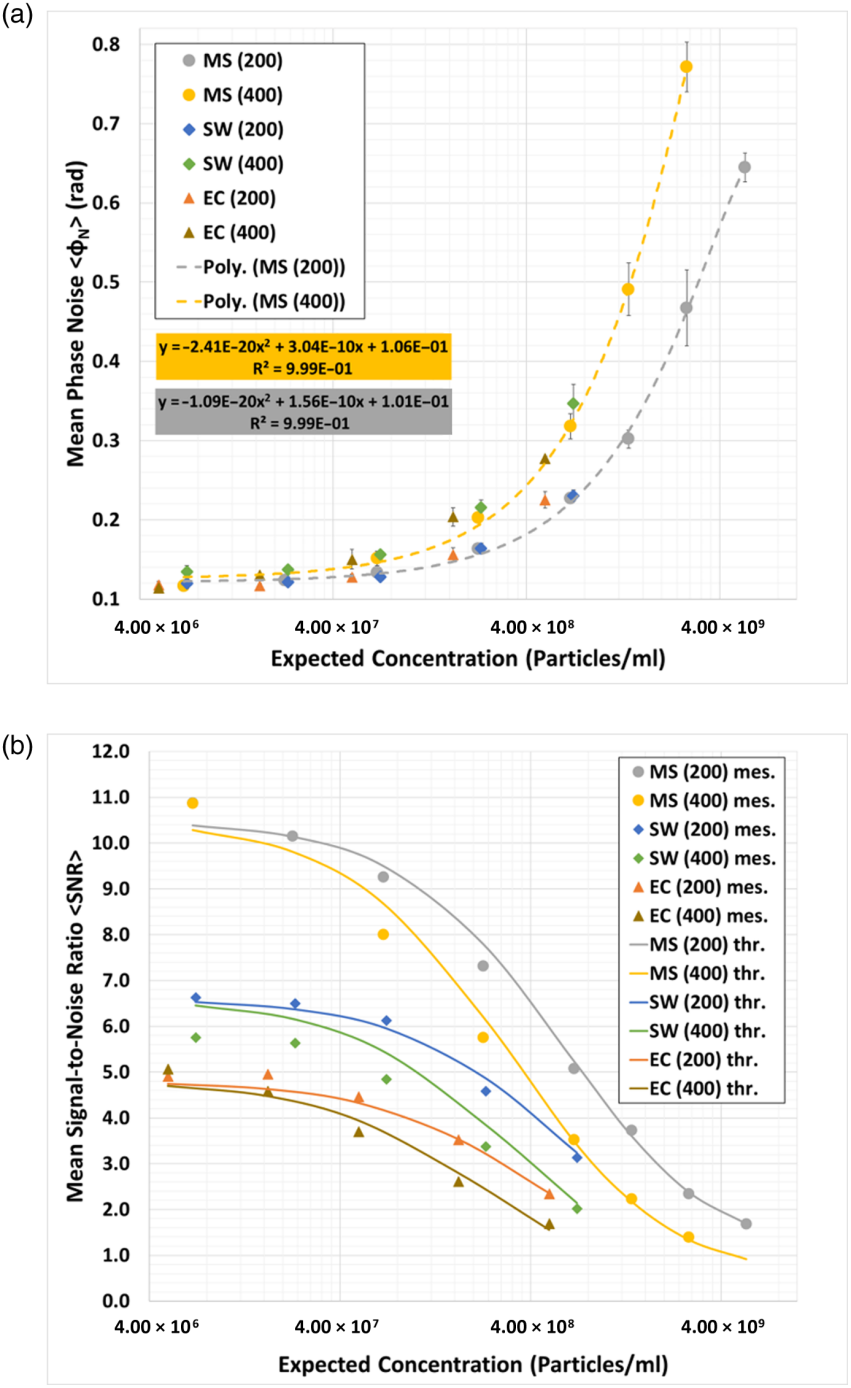
(a) Mean phase noise $\left\langle \phi_{N}\right\rangle$ calculated using Eq. (1) for various sample types, concentrations, and chamber heights. Dashed line corresponds to a quadratic interpolation of the values measured for microspheres. Interpolated polynomials are given in the color-coded boxes together with their respective coefficients of determination. The analysis includes three independent dilution series for microspheres and at least three biological replicates for bacteria, each captured at multiple positions within the same microfluidic chamber. The error bars represent one standard deviation of uncertainty. The dilutions 1:2400 and 1:10 for the 400  μm chamber height were omitted due to technical reasons; (b) mean signal-to-noise ratios for the various sample types, concentrations, and chamber heights. Points correspond to the ratio between the calculated phase signal $\left\langle \phi_{S}\right\rangle$ using Eq. (4) and the measured phase noise $\left\langle \phi_{N}\right\rangle$ using Eq. (1). The lines are the ratio between the calculated phase signal $\left\langle \phi_{S}\right\rangle$ using Eq. (4) and the interpolated phase noise ⟨ϕ^N⟩ in Eq. (2). MS, microspheres; SW, *S. warneri*; EC, *E. coli*; mes., measured, and thr., theoretical.

$\phi_{z}$ denotes the corrected and numerically refocused phase at a plane z and $\left\langle \phi_{z}\right\rangle$ its mean value. $N_{xy}$ refers to the total amount of pixels in a plane z, whereas $N_{z}$ is the total amount of numerically refocused planes from z to $z_{\max}$. By calculating $\left\langle \phi_{N}\right\rangle$ for each concentration, chamber height and sample type, as shown in Fig. 7(a), we notice a clear trend in the data and draw the following conclusions:

1. The sample type itself has negligible effect on $\left\langle \phi_{N}\right\rangle$.
2. $\left\langle \phi_{N}\right\rangle$ increases primarily with the concentration and chamber height.

This analysis answers the first question we proposed in Sec. 1 and highlights the effects of the concentration and chamber height as key parameters for influencing the measured particle count by increasing the background phase noise $\phi_{N}$.

Finally returning to the second question in Sec. 1, which states: Is it possible to analytically relate the parameters (sample type, concentration, and chamber height) to quantitatively predict a concentration range measurable with a given DHM system? We wish to answer this question by analytically deriving a mathematical expression for the SNR from the collected data. This SNR would give us an insight into the difficulty of the measurement problem in advance and would allow us to set initial limits on the measurable concentration range.

As we noted earlier, we consider that $\phi_{N}$ comprises two distinct contributions: particle dependent $\phi_{NS}$ and particle independent $\phi_{N0}$ noise sources. We now wish to somehow relate $\left\langle \phi_{N}\right\rangle$ to the concentration c in $\text{particles}/\mu m^{3}$ and chamber height L in μm using an analytical expression. We have found that by fitting the following mathematical expression:

⟨ϕ^N⟩(c,L,ϕN0)=ϕNS+ϕN0=L(k1c2+k2c)+ϕN0, (2)

from the microspheres data plotted in Fig. 7(a), we can estimate the fitting parameters $k_{1}$ and $k_{2}$ to be $-55\;\mathrm{rad}\;\mu m^{5}/{\left(\text{particles}\right)}^{2}$ and $0.78\;\mathrm{rad}\;\mu m^{2}/(\text{particles})$, respectively. ⟨ϕ^N⟩ represents the best least mean square fit to the data in Fig. 7(a), whereas $\phi_{N0}\approx 0.1\;\mathrm{rad}$ denotes the base-level phase noise measured using an empty chamber containing only the suspension medium.

We now turn our attention back to deriving an expression for estimating the SNR defined as

⟨SNR⟩=⟨ϕS⟩⟨ϕN⟩≈⟨ϕS⟩⟨ϕ^N⟩. (3)

$\left\langle \phi_{S}\right\rangle$ refers to an expression for the phase shift of a wavefront derived from theoretical principles. Assuming that the suspended particle has a thickness d (1  μm for microspheres and *S. warneri*^29^ and 0.73  μm for *E. coli*^30^) and a refractive index $n_{S}$ (1.388 for *S. warneri* and *E. coli*^31^ and 1.42 for microspheres^32^), it is possible to calculate an average phase signal strength $\left\langle \phi_{S}\right\rangle$ of a particle suspended in an aqueous solution with a refractive index of $n_{M}=1.333$ to be

$$\langle \phi_{S}\rangle (d,n_{S},n_{M},\lambda )=\frac{2\pi d(n_{S}-n_{M})}{\lambda },$$ (4)

with λ referring to the wavelength.^33^ Considering the assumptions made above, we calculate an average phase signal strength $\left\langle \phi_{S}\right\rangle$ of 1.05, 0.66, and 0.48 rad for microspheres, *S. warneri* and *E. coli*, respectively. These theoretical values match up with the measured phase signals of the numerically refocused particles in Figs. 3 and 5 quite well and allow us to determine the mean ⟨SNR⟩ in Eq. (3) of the numerically refocused phase images for each sample type, concentration, and chamber height by calculating the ratio between Eqs. (2) and (4)

⟨SNR⟩(d,nS,nM,λ,c,L,ϕN0)≈⟨ϕS⟩(d,nS,nM,λ)⟨ϕ^N⟩(c,L,ϕN0)=2πd(nS−nM)λ[L(k1c2+k2c)+ϕN0]. (5)

The calculated ⟨SNR⟩ using Eq. (5) matches up with the measurements quite well [Fig. 7(b)] and explains the large counting deviations in the case of *E. coli* as this sample type suffers from an inherently low ⟨SNR⟩ compared with *S. warneri* and microspheres.

## 5 Discussion

In this work, we propose a DHM system capable of counting bacteria over a wide range of concentrations. After presenting the numerical processing techniques in Sec. 2 and the optical setup in Sec. 3.1, we first quantitatively analyzed the numerical refocusing and resolving capabilities of the system using a USAF resolution target. Experiments in Sec. 4.1 confirmed an optical resolution of 1  μm over a numerical refocusing distance of 200  μm (Fig. 2). In addition, we characterized the counting capabilities of the proposed system using standardized microspheres of 1  μm diameter and microfluidic chambers of 200 and 400  μm chamber height. Here, we highlighted the strength of the measurable phase signal [Figs. 3(c1)–3(c2)] allowing the segmentation algorithm to distinguish singular particles within the numerically refocused volume [Figs. 4(a)–4(b)]. The concentrations measured with the proposed DHM system reflected the dilution steps quite well for both 200 and 400  μm chamber heights [Fig. 4(c)]. A CV of up to 20% was observed throughout the majority of the concentration range—see Fig. 4(d). Finally, we carried out counting experiments using *S. warneri* and *E. coli*. Analysis of the measurable phase showed varying signal strength ranging between 0.6 and 2 rad, depending on the bacteria type and orientation to the optical axis (Fig. 5). Bacteria that are oriented parallel to the optical axis (rows 1 and 4 of Fig. 5) exhibit a stronger phase signal compared with bacteria of the same kind oriented perpendicular to the optical axis (rows 2 and 3 of Fig. 5). The bacterial concentrations measured by numerically refocusing over the chamber height and counting segmented structures [Figs. 6(a)–6(d)] correlated well with the dilution steps between undiluted and 1:100 [Figs. 6(e)–6(f)]. This was noticeable for both bacteria types (*E. coli* and *S. warneri*) and chamber heights (200 and 400  μm). The deviations may be further minimized by using, e.g,. advanced AI-supported algorithms for segmentation and counting, which, however, were beyond the scope of the work presented here.

We continued to derive a model for predicting the SNR in the reconstructed phase image. Such a model allows for determining initial theoretical limits of the proposed system and for dimensioning the system parameters to better suit a given measurement problem. For example, we envision the demonstrated technology and proposed framework to be used as a method for phenotypic AST in microfluidic chambers. As this approach relies on single-cell detection of the bacterial growth and thus requires only small input cell numbers for the setup of an assay, it could help in reducing the time to result significantly when compared with state-of-the-art turbidity-based methods such as the bioMérieux VITEK 2^34^ or the BD Phoenix System,^35^ which are based on optical density measurements of bulk cell populations. Few single-cell detection methods have been commercialized for monitoring bacterial growth. The digital time-lapse microscopy-based oCelloScope system by BioSense Solutions^36^ and the phase-contrast-microscopy-based Quantamatrix dRAST system^37^ both use a conventional 96 plate format and a dedicated reader. The Accelerate Pheno System is based on fluorescence *in situ* hybridization of bacteria from blood cultures in cartridges.^38^ Baltekin et al.^39^ recently introduced a customized microfluidic chip, which allows for periodic counting/growth monitoring of rod-shaped bacteria in microchannels by 2D-phase contrast imaging. Although this system allows for the precise measurement of replication times of bacteria caught in those channels, the extraordinary small feature sizes of the microfluidic chip in the μm range may pose challenges for mass fabrication and thus might constitute a significant impediment to the scalability and cost efficiency of the method in routine use. Other QPI-based single-cell analysis workflows have been demonstrated to precisely quantify bacterial growth but requires either bacteria immobilization due to long acquisition times^6^ and/or do not allow for analyses in extended volumes.^7^ As the DHM-based workflow for bacteria counting proposed herein does not require any form of labeling, allows for rapid one-shot 3D imaging, and tolerates flexibility in cartridge formats, it offers potential advantages for use with lab-on-chip microfluidic devices in routine use. Our study seeks to support the use of DHM in such an approach by providing a framework to optimize assay-relevant parameters. In particular, using the formula presented in Eq. (5), we can determine the maximum measurable concentration for a given chamber height and required SNR. In addition, the formula assists in the manufacturing of microfluidic chips as it considers material-specific parameters such as surface roughness or material inhomogeneities, represented by the base-level phase noise $\phi_{N0}$.

Finally, to verify the SNR model’s accuracy, we attempted to predict previously reported SNR values of suspended bacteria captured with DHM. Bedrossian et al.^5^ empirically determined the SNR in the phase image of *Bacillus subtilis* suspended in 800  μm high chambers. Assuming a base-level phase noise $\phi_{N0}$ of 0.1 rad, a thickness of 1  μm and a refractive index of 1.388 for the bacteria, we were able to predict a constant SNR between $10^{5}$ and $10^{6}\;\text{cells}/\mathrm{mL}$ consistent with the results reported in the original research. At around $10^{6}\;\text{cells}/\mathrm{mL}$, the model predicts an initial drop in the SNR—similar to their measurement—reaching a decrease of −1.5  dB or a 15% drop at around $3\times 10^{7}\;\text{cells}/\mathrm{mL}$, close to the $10^{7}\;\text{cells}/\mathrm{mL}$ reported by Bedrossian et al.^5^ However, Bedrossian et al. report that the measured SNR maintains a constant value between $10^{7}$ and $10^{8}\;\text{cells}/\mathrm{mL}$, not decreasing below the initial drop of 15%, contrary to our model predicting a continued decrease of ca. 40% at around $10^{8}\;\text{cells}/\mathrm{mL}$. Other reports demonstrate a decrease in the SNR in the amplitude images as a result of multiple scattering at concentrations of $10^{8}\;\text{cells}/\mathrm{mL}$ compared with lower concentrations,^3^ which is more similar to our results. In addition, the slight deviations in our prediction and their reported concentration are probably the result of our assumption for $\phi_{N0}$ and their use of a DHM system with a different magnification and numerical aperture, thus having slightly different $k_{1}$ and $k_{2}$ values. In general, we expect that $k_{1}$ and $k_{2}$, which are empirically derived, may need to be calibrated for the optical system being used. We further note the work on this subject by Pu and Meng^40^ and Meng et al.,^41^ where they explicitly derive expressions for the SNR based on statistical analysis of scattering from multiple small particles, which has a dependence on the product of the particle density and the extent of the particle field along the optical axis. Specifically, they note in Ref. 40 a higher angular aperture of the imaging objective improves the information capacity of the hologram. These results were derived on the basis of assuming that scattering from a large number of scatterers follows a random process that can be described using zero-mean circularly complex Gaussian random variables.

## 6 Conclusion

In this work, we have presented an off-axis DHM system capable of counting microspheres (1  μm diameter), *S. warneri* and *E. coli* bacteria suspended in microfluidic chambers of up to 400  μm height. The employed post-processing techniques allowed the counting of particles in a single-shot manner and promised a lateral resolution of 1  μm. Experiments demonstrated the capability of the system to measure over a concentration range of $6.756\times 10^{6}$ to $5.405\times 10^{9}\;\text{microspheres}/\mathrm{mL}$ in the case of microspheres and over a dilution range of 1:100 to 1 (undiluted) of ${\mathrm{OD}}_{600}=1$ for *S. warneri* and *E. coli*. Finally, we derived an analytical function capable of predicting the SNR of suspended particles in the phase image based on their size, concentration, and the height of the chamber they occupy. In the future, we wish to analyze the scaling factors $k_{1}$ and $k_{2}$ in Eq. (2) and their dependency on various optical parameters such as the wavelength, magnification, and NA. We envision the proposed DHM system to be used in the clinic as an alternative to the conventional turbidity measurement as these systems can be built in a compact low cost manner with a high degree of robustness.^28^^,^^42^^–^^45^ In addition, the presented SNR model is expected to support the design and optimization of such systems by providing guidance based on specific measurement parameters.

## Supplementary Material

10.1117/1.JBO.30.10.106501.s1

10.1117/1.JBO.30.10.106501.s2

10.1117/1.JBO.30.10.106501.s3

10.1117/1.JBO.30.10.106501.s4

10.1117/1.JBO.30.10.106501.s5

10.1117/1.JBO.30.10.106501.s6

## Data Availability

Data underlying the results presented in this article are available upon a reasonable request to Hussein Kamel at hussein.kamel@ilm-ulm.de and Damien P. Kelly at damien.kelly@ilm-ulm.de.
